# Supplementary material for: Juniper Berry Oil as a Functional Additive in Chitosan–Water Kefiran–Paramylon Porous Sponges: Structural, Physicochemical, and Protein Interaction Insights
Source: Int J Mol Sci. 2025 May 31;26(11):5314. doi: 10.3390/ijms26115314 (PMC12155383; doi:10.3390/ijms26115314)
Supplement: Supplementary file 1 [file ijms-26-05314-s001.zip › ijms-3654052-supplementary.pdf]

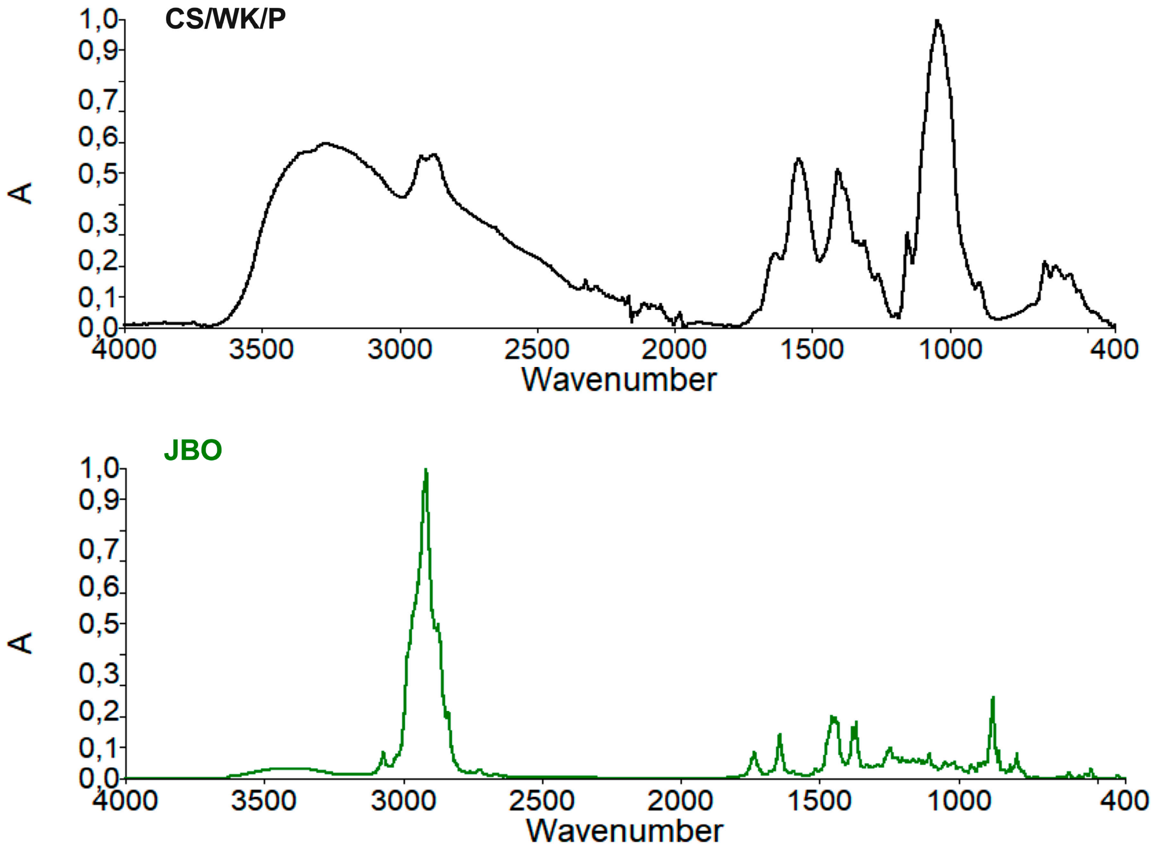

Figure S1. ATR-FTIR spectrum of the polysaccharide's mixture and juniper berry oil.

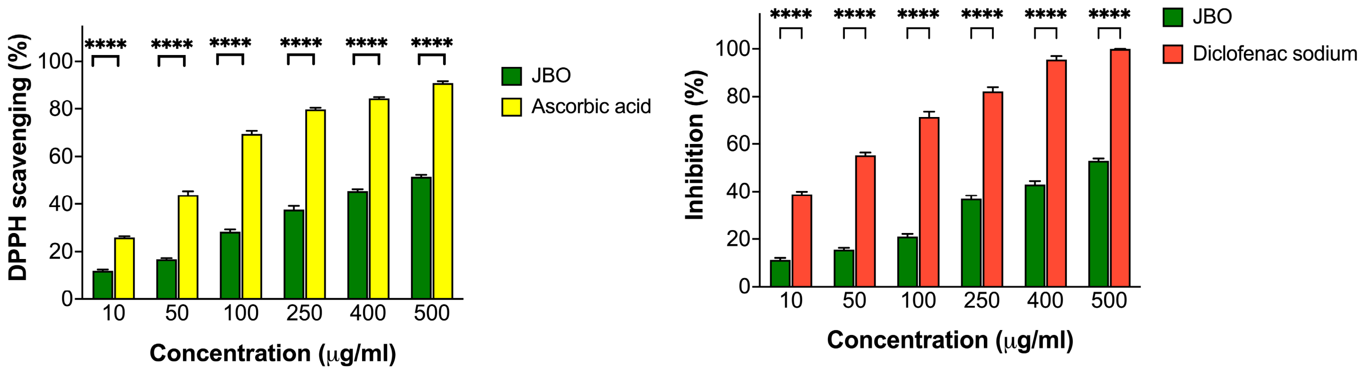

Figure S2. Antioxidant and anti-inflammatory properites of the juniper berry oil (JBO).

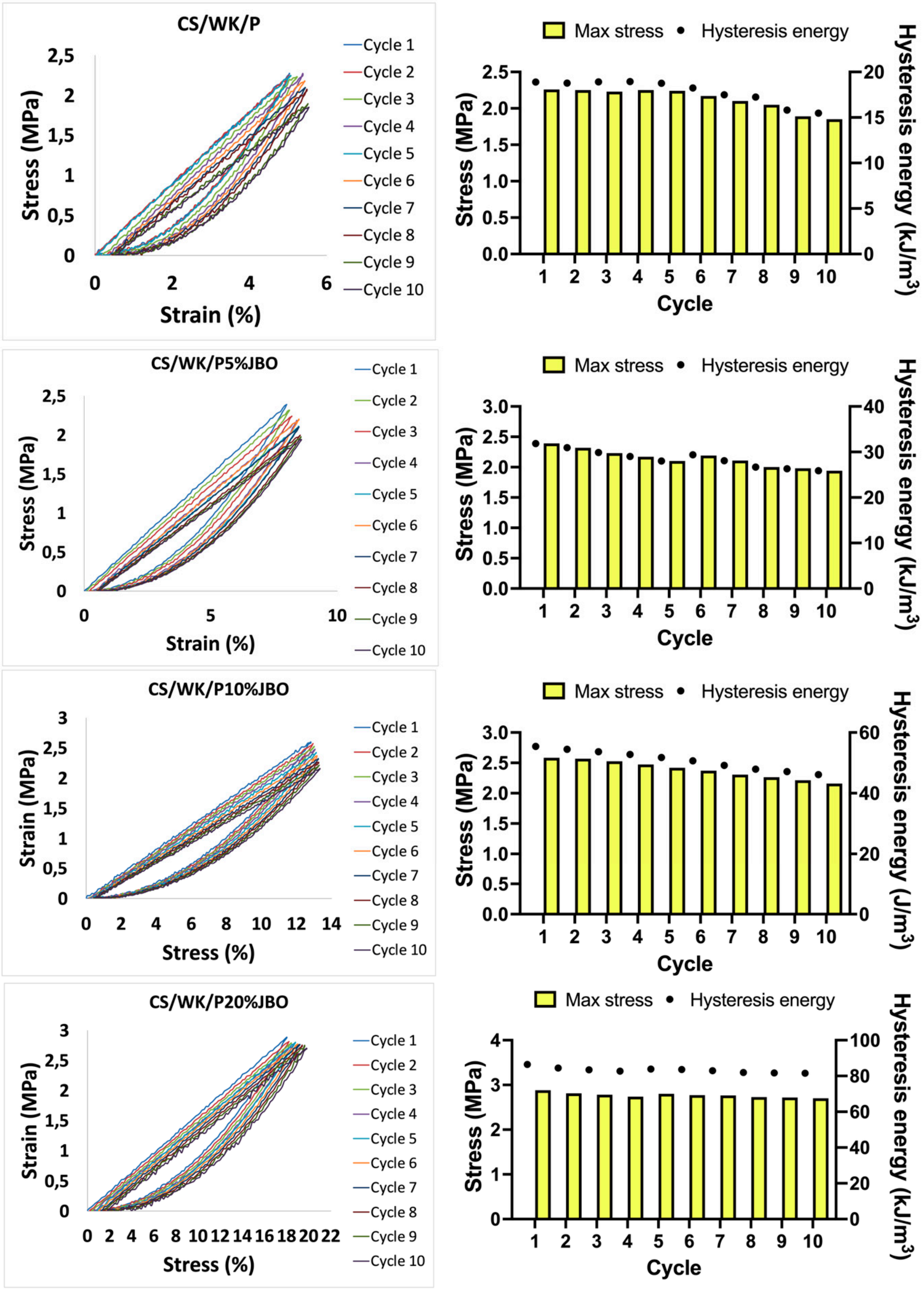

Figure S3. Mechanical cyclic performance of sponges. Strain–stress curves corresponding to the 1st and 10th loading–unloading cycles (A), and maximum stress and hysteresis energy recorded for the 1st and 10th cycles (B).
